# My secret: The social meaning of HIV/AIDS stigma

**DOI:** 10.1080/17290376.2014.932302

**Published:** 2014-07-01

**Authors:** N. Judgeo, K.P. Moalusi

**Affiliations:** ^a^ MA Industrial Psychology, a post-graduate student at the University of South Africa, is registered with the Health and Professions Council of South Africa as a Psychologist under the category Industrial, South Africa; ^b^ M.ADM Industrial Psychology, is a Senior Lecturer in the Department of Industrial & Organisational Psychology at the University of South Africa (UNISA), Pretoria, South Africa

**Keywords:** stigma, HIV/AIDS, people living with HIV/AIDS, qualitative research, Stigmatisation, VIH/SIDA, personnes vivant avec le VIH/SIDA, recherche qualitative

## Abstract

This study uses Goffman's [1963. Stigma: Notes on the Management of Spoiled Identity, New Jersey, Prentice-Hall] theory of stigma as an intellectual scaffold to help understand the social meaning of HIV/AIDS stigma from People Living with HIV/AIDS. The study adopts a qualitative approach because of its appropriateness for unravelling subjective phenomena such as the experiences of HIV/AIDS stigma. In-depth interviews were conducted with 10 HIV-positive employees of a retailing company located in the Western Cape province of South Africa who volunteered to participate in the study. The participants with the discreditable stigma internalised society's prejudice towards those living with the virus. As a result, the participants relied on self-isolation and social withdrawal to cope with enacted stigma. Managing information about one's status and deciding whether, who, when, etc., to tell are crucial questions. The participants feared being devalued by family, friends, co-workers and the community. In concurrence with Goffman [1963. Stigma: Notes on the Management of Spoiled Identity, New Jersey, Prentice-Hall] the HIV/AIDS stigma is seen as about relationships.

## Résumé

Dans cette étude, la théorie de la stigmatisation de Goffman (1963) sert d'échafaudage intellectuel pour contribuer à la compréhension de la signification sociale des tares associées au VIH/SIDA chez les personnes vivant avec le VIH/SIDA (PVVIH). Une approche qualitative a été adoptée car elle permet d'éclaircir les phénomènes subjectifs tels que les expériences de stigmatisation liées au VIH/SIDA. Des interviews en profondeur ont été menés auprès de dix employés séropositifs d'une compagnie de distribution située dans la province du Cap occidental en Afrique du Sud. Ceux-ci se sont portés volontaires pour participer à cette étude. Les participants victimes d'une stigmatisation déshonorante ont intériorisé les préjugés sociaux envers les personnes vivant avec le virus. En conséquence, les participants se sont tournés vers l'auto-isolement et le retrait social pour affronter cette stigmatisation. Gérer les informations relatives au statut sérologique et décider s'il faut le communiquer, à qui et quand etc. sont de questions cruciales. Les participants craignent d'être dévalorisés par leurs familles, amis, collègues et par leur communauté. En accord avec Goffman (1963), la stigmatisation du VIH/SIDA est perçue comme liée aux relations.

## Introduction

One of the major impediments to mending the scourge of HIV/AIDS is stigma (Feyissa, Abebe, Girma & Woldie 2012; Mahajan, Sayles, Patel, Remien, Ortiz, Szekeres, *et al*. 2008; Physicians for Human Rights 2011; UNAIDS 2007). Brown, Trujillo and Macintyre (2001) believed that the type and magnitude of people's reactions to this epidemic is largely due to HIV/AIDS stigma. HIV stigma remains the roadblock to a concerted action to reduce the transmission of HIV as it impedes prevention and treatment efforts (Campbell, Foulis, Maimane & Sibiya, 2005; Feyissa *et al*. 2012).

### Trends from the literature

In his seminal work, Goffman (1963: 3) defined stigma as ‘an attribute which is deeply discrediting, but it should be seen that a language of relationships, not attributes, is really needed’. Central to Goffman's (1963) notion of stigma is the issue of relationships. On its own, the attribute of stigma is neither creditable nor discreditable. As Goffman (1963) said, society labels an individual or a group as being deviant. The labelling happens in as diverse settings as abortion (Kumar, Hessini & Mitchell 2009), mental health (Stuart 2008), health-care workers who care for PLWHA (People Living with HIV/AIDS) (Sadoh, Fawole, Sadoh, Oladimeji & Sotiloye 2006) and families with children requiring mechanical ventilation (Carnevale 2007). In the eyes of the ‘normals’, the stigmatised person is seen as a ‘spoiled identity’ in that the person has a discrediting attribute and thus has to be treated with less respect. Individuals who are perceived as manifesting the discreditable attribute are tainted and devalued (Physicians for Human Rights 2011; Varas-Diaz, Serrano-Garcia & Toro-Alfonso 2005).

Deacon, Stephney and Prosalendis (2005) explained stigma as an ideology that identifies and links biological disease to negatively defined behaviour. In the context of HIV/AIDS, stigmatisation is explained as a process that devalues people infected with HIV (Miller & Forehand 2007). PLWHA are viewed as possessing the profoundly discrediting attribute.

The review of literature reveals that stigma is contextual (Herek & Glunt 1988; Norris, Besset, Steinberg, Kavanaugh, De Zordo & Becker 2011). For example, in America and most of Western Europe HIV/AIDS, stigma is associated with homosexuality and intravenous drug users whilst in South Africa heterosexual sex is regarded as the common mode of transmission (Campbell, Nair, Maimane & Sibiya 2008; Crandall 1991; Herek & Glunt 1988). However, in both instances, the concern is with what is regarded as socially sensitive issues.

HIV/AIDS stigma can have debilitating effects on the social life of the stigmatised. PLWHA experience enacted stigma and are subjected to a plethora of unpleasant treatment that includes discrimination, social ostracism and violence (Campbell *et al*. 2005; Herek, Capitanio & Widaman 2002). PLWHA have also been subjected to loss of resources such as customers and loss of jobs (Cao, Sullivan, Xu & Wu 2006). They are likely to experience stigma from some health-care providers who transmit stigma from the broader society and are also stigmatised in their communities (Andrewin & Chien 2008; Feyissa *et al*. 2012; Physicians for Human Rights 2011). HIV/AIDS stigma manifests in health-care facilities and poses a formidable hindrance to HIV/AIDS treatment (Health Policy Initiative, Task Order 1 2010; Nylblade, Stangl, Weiss & Ashburn 2009). Amongst others, low levels of education and irrational fears of HIV transmissions have been reported amongst health-care workers (Hossain & Kippex 2010).

From its nascent stage, the HIV/AIDS epidemic evoked negative and sometimes antagonistic attitudes towards PLWHA. In South Africa, the plight of the stigmatised is epitomised by the young Nkosi Johnson who was denied school admission, Gugu Dlamini who was attacked by a mob and murdered after she had publicly revealed her HIV/AIDS status, Lorna Mlofane who was raped and thereafter murdered after her three rapists discovered that she was HIV-positive (Skinner & Mfecane 2004; Stein 2003). The two latter incidents are a prominent feature of societies with pronounced power differences such as between men and women (Deacon *et al*. 2005; Physicians for Human Rights 2011). As Mbonu, van den Borne and De Vries (2010) noted, men and women who are HIV-positive or have AIDS may suffer from the same illness but society's predisposition to the assumed or subservient position of women may expose them to extreme negative responses. It can therefore be seen that stigmatisation is the process of devaluing because spoiled identities and undesirable differences do not exist naturally (Maluwa, Aggleton & Parker 2002). Enacted stigma, such as discrimination and prejudice, is viewed as just retribution for something the person or his/her parents did and is used to rationalise the treatment meted out at the stigmatised (Goffman 1963).

Goffman (1963: 4) distinguished between discredited and discreditable stigma. The former refers to a situation whereby ‘the stigmatised individual assumes that his differentness (personified by his HIV/AIDS positive status) is known about already or is evident on the spot’. Being discredited, the stigmatised has to manage the tension generated during social contacts and face prejudice. Discreditable stigma applies to a situation whereby the stigmatised assumes that his differentness in the form of HIV-positive status ‘is neither known by those present nor immediately perceivable by them’ (Goffman 1963: 4). The discreditable person has to manage information about his/her failing. However, Goffman believed that a stigmatised individual may experience both situations. The implication of this is that the stigmatised individual will respond to the social deviancy that others attribute to him/her or to his/her otherness. The stigmatised may also respond by creating their own social norms, alienating themselves psychologically and physically by way of keeping a distance from those that stigmatise them or they may engage in passing, so as to manage information about their failing in the presence of ‘normals’ (Carnevale 2007; Goffman 1963).

The concept of passing is central to Goffman's thesis of information control or stigma management. Discreditable individuals may use passing because they do not want to be seen to possess the failing. Germane to the subject of passing is the vital question of whether or not the failing is readily visible or perceptible. As Goffman said, visibility should be distinguished from its ‘known-about-ness’, ‘obtrusiveness’ and ‘perceived focus’. However, the concept of passing becomes irrelevant in instances where the failing is known to no one or is only known to the HIV-positive person. Discreditable individuals may pass to others as in the case of friends.

## About this study

The objective of this study is to explicate the experiences of HIV/AIDS stigma from PLWHA and how these experiences have affected their social life. Goffman's (1963) theory of stigma is used as an intellectual scaffold to explain these experiences.

## Methods

### Research strategy

This study adopts a qualitative approach because of its appropriateness for unravelling subjective phenomena such as the experiences of HIV/AIDS stigma. According to Pernice (1996), qualitative research strives to understand the unique experiences of the individual participants from their perspectives. Qualitative research is grounded on the interpretive and constructivist approach. From this perspective, truth is viewed as subjective reality which is constructed by the research participants. Qualitative research explores phenomenon under scrutiny by focusing on the participants. It endeavours to understand the social world, human experiences and social meaning.

### Research setting, entrée and ethical issues

The research was limited to HIV-positive employees of a retail company located in the Western Cape Province of South Africa. The company has branches in all the provinces of South Africa. It has HIV/AIDS policies and employs a professional nurse to perform the functions relating to the health of its employees. The nurse is registered with the Health Professions Council of South Africa and is therefore bound by the ethical code of the profession, specifically and germane to this study, nurse–patient confidentiality.

The organisation granted permission to conduct the study. However, only the company nurse officially knew which employees were HIV-positive. A letter was written to the company healthcare provider or nurse containing sufficient information about the research and soliciting the participation of the respondents. Therefore, access to the participants was gained indirectly through the company nurse who assisted the research by conveying the objectives of the study to the potential participants and the request that they participate. The company nurse discussed ethical issues, such as anonymity, confidentiality and withdrawal at any stage of the research, with the potential participants. They were also informed of their right to refuse to answer some of the questions without prejudice. For instance, the participants preferred not to sign the consent forms as this might enable others to identify them and insisted that verbal permission was sufficient.

An ethical clearance was obtained from the University's Ethics Research Committee.

### Sample

By and large, qualitative inquiry works with small samples of participants that are selected purposefully to permit understanding of a phenomenon in depth (Pernice 1996). The issue of the small sample has implications to the generalisability of the results. The thrust of qualitative studies is neither the numerical distribution of study participants among factors nor the number of people that hold a particular point of view but on how and why people believe the way they do. The intention is therefore not to generalise the results to a bigger population (Brown 1980).

It was not possible to identify even the number of PLWHA in the organisation because of the sensitivity of the information. The identity of individuals who were HIV-positive was only disclosed and known to the company healthcare provider who could not reveal the number of employees who were HIV-positive. It was therefore not possible to identify the number of those who declined or did not volunteer. In short, a list of HIV-positive employees from which individual participants could be drawn was non-existent. However, accurate information regarding the criterion for participating (HIV-positive) was provided to the company healthcare provider. Ten employees volunteered to participate. All 10 were full-time employees of the organisation.

Pseudonyms and brief descriptions of the characteristics of the participants are provided.

Participant 1 identified as Dan is a single black male in his twenties who contracted the virus almost six years prior data collection. He was only 22 years old and described himself as innocent. He reported that he did not understand what HIV/AIDS was then.

Participant 2 alias Nomsa is a single black female in her twenties. She was diagnosed HIV-positive two years prior data collection.

Participant 3 named Brian is a coloured male in his thirties who was diagnosed HIV-positive three years prior data collection. He is separated from his wife.

Participant 4 named Dina is a black female in her thirties and was diagnosed HIV-positive 10 years prior data collection.

Participant 5 alias Joy is a black single female in her twenties. She was diagnosed HIV-positive four years prior the data collection.

Participant 6 code-named Peter is a black male in his twenties. He contracted HIV approximately four years prior data collection. He had a girlfriend but they were not intimate because he did not want to infect her with the virus. He was looking for a long-term relationship and thought he had found a wife in his girlfriend. At a point when he thought it was time to take their relationship to a new level, he revealed his HIV-positive status to her. His girlfriend left him after he had disclosed his HIV-positive status.

Participant 7 referred to as Pretty is a single coloured female in her twenties. She was 19 years old when she discovered that she was HIV-positive. She was repeatedly raped by her uncle when she was 12 years old. In the absence of her father, the uncle had assisted the family financially. She broke her silence and revealed her HIV-positive status and the repeated rapes by the uncle over the years to her mother. However, the mother accused her of sleeping with many boys at school and chased her away.

Participant 8 named Mildred is a divorcee coloured female in her forties. She was diagnosed HIV-positive eight years prior the interview. Her husband was abusive and became alcoholic. He was admitted into a rehabilitation centre for treatment. She discovered her HIV-positive status after he revealed to her that he was HIV-positive.

Participant 9 called Rodney is a male coloured in his forties. He was diagnosed HIV-positive 10 years prior the interview.

Participant 10 named Francis is a female coloured in her twenties. She was diagnosed HIV-positive four years prior the interview. Her husband married her knowing her HIV-positive status.

### Data collection

A semi-structured interview schedule was used to conduct one-on-one in-depth interviews with the participants. In addition to biographical questions, the interview guide also comprised open-ended items about the participants' personal experiences of being HIV-positive (e.g. do you consider your life has changed due to your HIV-positive status? How has being HIV-positive affected your relations with friends, family, co-workers and the community? The interview guide also included questions about whether the participants felt comfortable revealing and whether they have revealed their HIV-positive status; instances when they felt stigmatised and how they dealt with stigmatising situations. The data were recorded by way of writing the participants' verbatim statements as they did not approve of audio recording. This resulted in lengthy interviews as attempts were made to capture the responses verbatim.

### Data analysis

Data were collected and analysed by the first author. To minimise bias and enhance the rigour of the study, the second author immersed himself into the data by reading the raw data and re-examining the first author's preliminary analysis. The final analysis was the outcome of the authors' consensus.

The data analysis followed in this study was thematic content analysis following Aronson (1994) and Braun and Clarke (2006). Data immersion and familiarisation were followed by identifying the patterns of respondents' experiences through their direct quotes. This was followed by coding the data by way of grouping all data that relate to the already classified patterns (Braun & Clarke 2006). Patterns were collated and combined into potential themes. Themes bring together components or fragments of ideas or experiences, which are meaningless when viewed alone (Aronson 1994). Themes become apparent from the participants' stories as transcribed from the interviews. The participants' experiences of HIV/AIDS stigma are pieced together to form a comprehensive picture. An effort was made to refine the specifics of each theme and the overall story the analysis tells, generating names for each theme. The literature was interwoven in the themes.

## Findings

The findings report on how the participants as both discredited and discreditable individuals experience HIV/AIDS stigma. These experiences are reported according to themes and the respondents' verbatim statements are used to illuminate the stories behind their responses.

### The attribute spoils the identity

The respondents revealed that their contraction of HIV/AIDS heralded an adverse change in their lives. While a few of them reported physical decline, all the respondents revealed how the contraction redefined their social life. Central to this is what the respondents regard as the stigmatising individuals' negative definition of the behaviour of PLWHA. Friends, family, the workplace and the society in general tend to view HIV-positive people as irresponsible, filthy and immoral. Brian reported as follows:

I think they feel that we are dirty people because if you are clean, like a virgin or only have one partner your whole life, then you would not get it. Also, people think we will die soon and therefore they must stay away from us because we are angry and may pass it on to them. That whole thing that went on a few years about pricking people with needles that had HIV blood on it has scared people. They think we are all cruel and angry and wanting to infect others because we are dying. I just know that generally, people rather not deal with us and avoid us if they know we are HIV positive.

Nomsa's experience captures the negative definition of the behaviour of PLWHA by the ‘normals’:

People believe that if you have HIV/AIDS then you deserve it because maybe you slept with more than one person. They think you were irresponsible and immature.

The reports that the respondents hear about the behaviour of HIV-positive people tend to suggest that heterosexual sex is the primary mode of HIV transmission. Nomsa revealed that:

I hear how they openly speak about people who are HIV-positive. They laugh at them and say that they are irresponsible. Like don't they know how to use a condom or that they are prostitutes sleeping with everyone, so they deserve it. I listened to them and know that they will not be able to accept me being [HIV] positive.

Brian reported the experience of his neighbour who revealed that her husband was HIV-positive:

No one visited him. Everyone spoke about him and how sad it was. Some said he deserved it because he was a real player and slept around. Others said that if they went near him or went to see him, then they could get it as well because they would be in the same room. When he died, like 2 months later, no one went to his funeral. His wife buried him alone and she still stays in that house. She has nowhere else to go. People don't talk to her right anymore as well. They like gossip all the time. Once you live with someone who has HIV, people think you have it too.

Francis, who was raped and as a result became infected with HIV, stated:

When I was raped and everyone knew about it in the neighbourhood, people treated me differently. They used to say shame that poor girl or she must have deserved it because she should not have let him in the house wearing that mini skirt. They said horrible things about me and treated me like I had done something wrong and I had to live with it. If they [the neighbourhood community] knew I was HIV-positive as well, I can only imagine the things people would gossip about. I would not be able to handle it.

An HIV-positive person can no longer be offered the respect and recognition that the uncontaminated aspects of his/her social identity have led him/her to anticipate receiving. Some of the respondents tend to think that whatever others profess, they do not really accept PLWHA on equal grounds. Joy's workplace experiences resonate with this statement:

Managers can do something like say we are not promoting you because you still need development in that area. But the real reason they are not giving you the job is because I have HIV/AIDS. In this way they work around the policy, still covered but can still deny me my rights. I rather not take any chances.

Rodney shared a similar experience:

I have told my line manager that I was living with HIV/AIDS and I wanted him to know in case I get sick. But soon all the managers knew. The managers started giving me lesser work and some staff members began avoiding me.

So in the eyes of the others, the stigmatised person is seen as ‘spoiled identity’ in that the person has a discrediting attribute.

### HIV/AIDS stigma has a negative impact on relationships

Some of the participants expressed the view that some people view HIV/AIDS as highly infectious and that people who are infected should be avoided. People are not interested in knowing more about HIV/AIDS. Dina expressed the view that all the ‘normals’ seemed to care about is that HIV-positive people possess some undesired differentness and should be avoided:

I think people fear us and will rather stay away and pretend we do not exist. I think that most people do not want to associate with us or form relationships with us because they are afraid that we will infect them. They want us to stay away from their worlds, because we are infected and we could infect their world.

Peter's expressions revealed society's unwillingness to grapple with HIV/AIDS-related issues:

I think people are just scared to know more about this [HIV/AIDS]. People [would] rather not hear about it because then they do not have to deal with it.

It is not uncommon for PLWHA to experience rejection by family, friends and the larger community. Friends may not always welcome the news. Joy reported:

I did reveal my status to my friends once, people I thought were my friends but that turned out badly. They just stopped being my friends, isolated me. So from that time I have not revealed to anyone except the counselling group that I attend.

The negative response may also come from people who are related to the stigmatised individual through social structures, such as one's parents, sons or daughters. Some respondents who experienced family rejection responded. Pretty, who had been repeatedly raped by her uncle from an early age, articulated her experience:

My family threw me aside. No support, no one to lean on when I can't go on anymore. If you have someone to support you, no matter what you are living with, you will find the willpower to carry on.

Peter also experienced rejection. He had been in a relationship with her girlfriend but they were never intimate. He dreamt of a long-term relationship between them:

I had to tell her about my status and I sat her down and explained how much I love her and how I see myself marrying. I told her that I was HIV positive and that even with the virus, I will never get her infected. We will take all the precautions to be safe. She was not happy. She said that she could not live with someone that is HIV positive and that she is too young to take on the responsibility. We spoke for hours, I think till the next morning but she ended it with me. Terrible, it hurt more than when I found out I was HIV positive. I loved her and thought that our love would bring us together no matter what. But I guess that was not true. So that was the last time I was in a relationship with someone.

An HIV-positive individual's interaction with Goffman's (1963) ‘normals’ may be stressful up to the point where the individual self-isolates. Pretty and Nomsa's respective statements captured this aptly:

I don't really have friends. I live a lonely life. It is still hard telling people what I have gone through and what I have to live with.

I chose not to have a boyfriend because I am positive. I think it would be difficult to start a relationship with someone because I will be putting them at risk of infection. Plus it will be difficult being with someone because I will always wonder when I will get sick and I am a burden to that person.

Pretty went to stay with her grandmother who lived in the same street as her mother and uncle. When she reported the uncle's abuse, her mother thought she was lying about her uncle's alleged abuse. After finding herself a job and a flat in town, she would send her grandmother money every month but:

I never want to go home, that is my past and I am leaving it behind me.

However, some of the respondents revealed that they had significant others who were sympathetic to their situation. These were people who played a pivotal role in the life of the participants. Joy revealed that she sought social support by joining the centre that gives counselling to PLWHA:

I have joined a counselling centre for people living with HIV/AIDS.

The sympathetic others may also come from one's friends and own family as in the case of Brian:

Telling my family had positive effects on me. They have helped me a great deal. I am just not ready telling other people.

Dina, who concealed her HIV-positive status for a year, finally revealed to her status:

I trusted a very close friend and family member. They have supported me. But not everyone is like that or has the same attitude. I feel that generally, people will be scared of you and will want to stay away. They can get angry at you and say it was my fault I am HIV-positive and I deserve it.

### Managing stigma: To tell or not to tell

Two serious issues with ominous implications for HIV-positive individuals are, first, how to manage information about one's own failing and second, how to manage tension when social contacts are made. Some discreditable individuals coped by using non-disclosure; others who are discredited asserted their fundamental right to be treated with respect and human dignity.

A discreditable person may conceal information about his/her HIV-positive status because such information is discrediting about the self. Nomsa provides an indication of the gravity of the issue of disclosure when reporting that he/she has not even revealed his/her status to family members because of fear of their response:

I have not told my family about being positive. I think they will not understand me and how I got myself in this mess. I don't want to be blamed and treated differently by my family. I have decided to keep this to myself.

Sometimes PLWHA do not reveal their HIV/AIDS status because such disclosures may have serious consequences for the entire family. Dan describes the potential danger that accompanies disclosure:

It will bring shame on my family because people with this disease like me are not accepted in the community. We are looked at as being dirty and unclean. Instead of just kicking me out, the community may even kick out my entire family and I can't let that happen.

The devaluing of the lives of PLWHA is not limited to family and friends. The wrath of the larger community may be extreme and elicits fear that compels the stigmatised not to disclose their status. PLWHA face a lot of enacted stigma such as discrimination and prejudice after disclosing their status. The prejudice may come from family or others socially distant from the person being stigmatised as observed by Pretty who recounted the experience of a young neighbour who committed suicide. When asked why the boy committed suicide, she asked the interviewer if she knew the shame and guilt you live with when you carry this [HIV]:

Where I lived, there was a boy that committed suicide. We all knew he had AIDS. He was open about it and people did not accept it. They were cruel and hurtful. The old people would say he was filthy and unclean. The younger boys would beat him. His family was ashamed of him. He thought killing himself would be better than living with the shame.

The discredited may sometimes assert their human dignity and stand their ground or may simply walk away from the situation. The challenges are different for individuals whose differentness is apparent. Some PLWHA such as Francis have resorted to leaving the social setting rather than face prejudice:

I think walking away from the situation will help it from going out of control. I have never dealt directly with discrimination because of my status. I think walking away and not getting yourself harmed or humiliated is better than standing there while someone argues with you and makes you feel small.

Others like Mildred would assert their right if faced with stigmatising situations:

I guess if someone did discriminate against me because of being HIV-positive, I would get very angry. I may just give them [a] piece of my mind and set them straight.

I would stand my ground and tell them that what they are saying or doing is wrong and they are going against my human rights. If they become physical I would try and walk away but to be fair to myself I will have to stand up for my rights.

The preceding findings reveal that the discredited and the discreditable may employ different ways of coping with stigma.

## Discussion

One of the central theses of Goffman's stigma theory is that society classifies individuals into ‘normals’ and ‘deviants’. This classification is ideological (Deacon *et al*. 2005; Goffman 1963). The people regarded as ‘deviants’ are stigmatised because they are said to be discounted and tainted, possessing an undesired differentness from what the ‘normals’ anticipated. The stigmatising constructs an ideology or a stigma theory that rationalises the treatment meted out to PLWHA by the ‘normals’. The attribute that they possess disqualifies them from full acceptance by the ‘normals’ (Mahajan *et al*. 2008; Oyserman & Swim 2001).

Society links the presence of the biological disease agent with negatively defined behaviours. In the USA, HIV/AIDS is associated with homosexuality and intravenous drug users, while in South Africa promiscuous heterosexual sex is perceived as the primary mode of transmission. Attributing stigmatised medical conditions such as HIV/AIDS to lack of personal responsibility has the effect of distancing the ‘moral majority’ from risk (Deacon *et al*. 2005). Crandall (1991) reported that some ways of becoming infected with HIV/AIDS are more stigmatised than others. The strength of the stigma increases when the cause of HIV infection is seen as some avoidable and negative behaviour such as promiscuity or other morally sensitive issues. The reverse is true when the HIV infection is thought to be accidental such as due to blood transfusion. The response is expected to be pity. However, the fact about HIV infection is that multiple partners and promiscuity may increase the chances of contracting it but it can be contracted by a person with one unfaithful partner (Deacon *et al*. 2005; Tanga, Atuyambe, Murphy, Ringheim & Woldehanna 2007).

Individuals have long been socialised into knowing what it means to be ‘normal’ and to be HIV-positive or have AIDS. Individuals come to know that the society tends to treat those who are HIV-positive or have AIDS with less respect. This knowledge may make it easier for PLWHA to uncover the reality behind the façade that the ‘normals’ profess. As Goffman (1963: 7) said, the stigmatised ‘may perceive, usually quite correctly, that whatever others profess, they do not really ‘accept’ him and are not ready to make contact with him on equal grounds’. The stigmatised can no longer be accorded the respect and recognition that ‘the uncontaminated aspects of his social identity have led them to anticipate extending, and have led him to anticipate receiving’ (Goffman 1963: 8–9). Through stigma, the life chances of the stigmatised are reduced. For instance, the career prospects of the stigmatised are curtailed by the differentness or the fact of being HIV-positive. This sentiment is echoed by some of the respondents regarding the diminished chances for promotion or development that they experienced in their company.

Central to Goffman's (1963) notion of stigma is the issue of relationships. Stigma is not just an attribute. It should be seen that a language of relationships is needed. Such a language involves the labelling of a person as discredited or deviant. The negative labelling reaffirms the normalcy of the person doing the labelling (Alonzo & Reynolds 1995). The results of the study authenticate the notion that stigma is a social process that can be enacted by and experienced from friends, family, the workplace and broader society.

There are instances when a discrepancy develops between an individual's virtual and actual identity. Actual identity is about the attributes that the person actually possesses whilst virtual identity concerns those that society imputes to the person. When the discrepancy is known about, it may spoil the social identity of the stigmatised. Social identity entails the attributes that the person is thought to possess in relation to others. The effect of stigma is alienation from oneself and society and facing an unaccepting world.

In line with Deacon *et al*. (2005), the results revealed that when the ‘normals’ express stigmatising thoughts, even if indirectly, PLWHA tend to experience the moral judgement projected onto them. The respondents expressed the view that the contraction of HIV spoiled their identity. The direct and indirect experiences of stigmatising views from the ‘normals’ may reveal the internalisation of stigma by some of the participants. In responding to stigma, the individual may self-stigmatise by way of accepting the negative social judgements of their own identity.

Within the social milieu of the stigmatised individual, Goffman (1963) identified two categories of sympathetic others, namely, the ‘own’ and the ‘wise’. The ‘own’ are people who share the stigma with the stigmatised person. The ‘wise’ are

persons who are normal but whose special situation has made them intimately privy to the secret life of the stigmatised individual and sympathetic with it, and who find themselves accorded a measure of acceptance, a measure of courtesy membership in the clan.

The presence of the wise presents the stigmatised with an accepting milieu or occasion whereby he/she does not have to feel ashamed in spite of his/her differentness. In this study the wise person is the nurse who was trusted and provided the participants with vital medical service. However, research revealed that healthcare providers suffer from HIV/AIDS stigma and that they do associate PLWHA with this stigma (Hossain & Kippex 2010; Physicians for Human Rights 2011).

The other type of wise person is the individual who is related through social structure to a stigmatised individual, such as a son or daughter of an HIV-positive person. In fact, stigma can spread from the stigmatised individual to those with close connections to him/her as society's tendency is to treat them the same. Some of the participants expressed the view that if they revealed their HIV-positive status, the community may victimise their family: ‘Instead of just kicking me out, the community may even kick out my entire family and I can't let that happen’.

Goffman believed that a stigmatised individual may have experience of both discredited and discreditable stigma. Regarding being discredited, the stigmatised has to manage the tension generated during social contacts and face prejudice against PLWHA, whilst the discreditable has to manage information about his/her failing.

The study has revealed that individuals cope with HIV/AIDS stigma differently. For some individuals, particularly the discreditable, non-disclosure of one's status is an effective way of managing stigma. However, this non-disclosure is not denial of one's HIV-positive or AIDS status. It entails disclosure to health care providers, such as the nursing sister in the study, and it may not mean non-disclosure to one's intimate or sexual partners. On the contrary, it may mean abiding by safer sex practices as in the case of the respondent who informed her partner and the one who preferred not to have a boyfriend. These individuals may not disclose their status because they want privacy, piece of mind and want to avoid being stigmatised. PLWHA encounter stigma from the media and the ‘normals’. Non-disclosure may denote that they have internalised society's prejudice towards those living with the virus. As a result, some of the participants relied on self-isolation and social withdrawal to cope.

Managing information about one's status and deciding whether, who and when to tell are crucial questions. The discreditable might have sought to manage information and prefer not to personally experience stigmatising situations (Deacon *et al*. 2005). However, Campbell *et al*. (2005) reported how the social context may obscure or even preclude younger people from testing and disclosing their HIV status and thereby increase the transmission through heterosexual sex. The negative attitudes of the health care workers exacerbate the desolate situation facing PLWHA (Sadoh *et al*. 2006; Sadoh, Sadoh, Fawole, Oladimeji & Sotiloye 2009).

It has also been revealed from the results of the study that some PLWHA are very assertive of their human rights. PLWHA are often subjected to unfair discrimination, such as not being hired or promoted or even offered training and development opportunities because of the perception that they are infectious and/or that they may die soon (Deacon *et al*. 2005; Physicians for Human Rights 2011).

## Conclusion

The results of the study clearly reveal how HIV/AIDS stigma is experienced by the participants. Spoiled identity, social relations and the need to manage information emerged as strong themes in this study. Stigma is about social relations. Linking HIV/AIDS with immorality or aberrant lifestyle has the effect of creating in-group and out-group categories or ‘we are HIV-negative’ and ‘they are HIV-positive’ groups. For the self-stigmatising HIV-positive person the consequence is facing a less accepting world that includes rejection by family, friends and society. This may entice a person not disclose one's serostatus, to self-isolate and not seek support group, HIV/AIDS treatment and the possibility of engaging in unprotected sex. However, the results of the study may suggest that concealing one's status was not a ploy to engage in unsafe sex. The study points out the urgent need to scale up the response to combat HIV/AIDS stigma through actions that are rooted in the experiences of PLWHA. The scaling up of anti-retroviral treatment, the attitude of the health-care providers and social action and mobilisation have a fundamental role in curtailing HIV/AIDS stigma.

## References

[CIT0001] Alonzo A., Reynolds N. (1995). An Exploration and Elaboration of a Stigma Trajectory. Social Science and Medicine.

[CIT0002] Andrewin A., Chien L. Y. (2008). Stigmatisation of Patients with HIV/AIDS among Doctors and Nurses in Belize. AIDS Patient Care and STDs.

[CIT0003] Aronson J. (1994). A Pragmatic View of Thematic Analysis. The Qualitative Report.

[CIT0004] Braun V., Clarke V. (2006). Using Thematic Analysis in Psychology. Qualitative Research in Psychology.

[CIT0005] Brown S. R. (1980). Political Subjectivity.

[CIT0006] Brown L., Trujillo L., Macintyre K. (2001). Intervention to Reduce Stigma: What have We Learned? Horizons.

[CIT0007] Campbell C., Foulis C. A., Maimane S., Sibiya Z. (2005). I have an Evil Child at My House: Stigma and HIV/AIDS Management in a South African Community. American Journal of Public Health.

[CIT0008] Campbell C., Nair Y., Maimane S., Sibiya Z. (2008). Supporting People with AIDS and their Careers in Rural South Africa: Possibilities and Challenges. Health and Place.

[CIT0009] Cao X., Sullivan S. G., Xu J., Wu Z. (2006). Understanding HIV-related Stigma and Discrimination in a “Blameless” Population. Aids Education and Prevention.

[CIT0010] Carnevale F. A. (2007). Revisiting Goffman's Stigma: The Social Experience of Families with Children Requiring Mechanical Ventilation at Home. Journal of Child Health Care.

[CIT0011] Crandall C. S. (1991). Multiple Stigma and AIDS: Illness Stigma and Attitudes toward Homosexuals and IV Drug Users in AIDS-related Stigmatisation. Journal of Community and Applied Social Psychology.

[CIT0012] Deacon H., Stephney I., Prosalendis S. (2005). Understanding HIV/AIDS Stigma: A Theoretical and Methodological Analysis.

[CIT0013] Feyissa G. T., Abebe L., Girma E., Woldie M. (2012). Stigma and Discrimination against People Living with HIV by Healthcare Providers, Southwest Ethiopia. BMC Public Health.

[CIT0014] Goffman E. (1963). Stigma: Notes on the Management of Spoiled Identity.

[CIT0015] Health Policy Initiative, Task Order 1 (2010). Measuring the Degree of HIV-related: Stigma and Discrimination in Health Facilities and Providers: Working Report.

[CIT0016] Herek G. M., Capitanio J. P., Widaman K. F. (2002). HIV-related Stigma and Knowledge in the United States: Prevalence and Trends, 1991–1999. Journal of Public Health.

[CIT0017] Herek G. M., Glunt E. K. (1988). An Epidemic of Stigma: Public Reactions to AIDS. American Psychologist.

[CIT0019] Hossain M. B., Kippex S. (2010). HIV-Related Discriminatory Attitudes of Healthcare Workers in Bangladesh. Journal of Health Population Nutrition.

[CIT0020] Kumar A., Hessini L., Mitchell E. M. (2009). Conceptualising Abortion Stigma. Culture, Health and Sexuality.

[CIT0021] Mahajan A. P., Sayles J. N., Patel V. A., Remien R. H., Ortiz D., Szekeres G., et al. (2008). Stigma in the HIV/AIDS Epidemic: A Review of the Literature and Recommendations for the Way Forward. AIDS.

[CIT0022] Maluwa M., Aggleton P., Parker R. (2002). HIV and AIDS-related Stigma, Discrimination, and Human Rights: A Critical Overview. Health and Human Rights.

[CIT0024] Mbonu N. C., van den Borne B., De Vries N. K. (2010). Gender-related Power Differences, Beliefs and Reactions Towards People Living with HIV/AIDS: An Urban Study in Nigeria. BMC Public Health.

[CIT0026] Miller C., Forehand R. (2007). Measurement of Stigma in People with HIV: A Re-examination of HIV Stigma Scale. Journal of AIDS Education and Prevention.

[CIT0027] Norris A., Besset D., Steinberg J. R., Kavanaugh M. L., De Zordo S., Becker D. (2011). Abortion Stigma: A Reconceptualization of Constituents, Causes and Consequences. Women's Health Issues.

[CIT0028] Nylblade L., Stangl A., Weiss E., Ashburn K. (2009). Combating Stigma in Health Care Settings: What Works?. Journal of the International AIDS Society.

[CIT0029] Oyserman D., Swim J. K. (2001). Stigma: An Insider's View. Journal of Social Issues.

[CIT0030] Pernice R. (1996). Methodological Issues in Unemployment Research. Journal of Occupational and Organisational Psychology.

[CIT0031] Physicians for Human Rights (2011). Ensuring Equality: A Guide to Addressing and Eliminating Stigma and Discrimination in the Health Sector.

[CIT0032] Sadoh A. E., Fawole A. O., Sadoh W. E., Oladimeji A., Sotiloye O. S. (2006). Attitudes of Healthcare Workers to HIV/AIDS. African Journal of Reproductive Health.

[CIT0033] Sadoh A. E., Sadoh W. E., Fawole A. O., Oladimeji A., Sotiloye O (2009). Attitude of Health Care Workers to Patients and Colleagues Infected with Human Immunodeficiency Virus. Journal of Social Aspects of HIV/AID.

[CIT0034] Skinner D., Mfecane S. (2004). Stigma, Discrimination and the Implications for People Living with HIV/AIDS in SA. Journal of Social Aspects of HIV/AIDS.

[CIT0035] Stein J. (2003). HIV/AIDS Stigma: The Latest Dirty Secret.

[CIT0036] Stuart H. (2008). Fighting the Stigma Caused by Mental Disorders: Past Perspectives, Present Activities, and Future Directions. World Psychiatry.

[CIT0037] Tanga E. O., Atuyambe L., Murphy C. K., Ringheim K. E., Woldehanna S. (2007). Examining the Actions of Faith-based Organisations and their Influence on HIV/AIDS-related Stigma: A Case of Uganda. African Health Sciences.

[CIT0039] UNAIDS (2007). http://www.unaids.org.

[CIT0040] Varas-Diaz N., Serrano-Garcia I., Toro-Alfonso J. (2005). Aids Related Stigma and Social Interaction: Puerto Ricans Living with HIV/AIDS. Journal of Qualitative Health, Research.

